# Transplantation Beyond Species: The Present and Future of Xenotransplantation

**DOI:** 10.1590/1516-3180.2025.1435.15072025

**Published:** 2025-10-06

**Authors:** Luiz Fernando Caneo, Tadeu Thomé, Paulo Manuel Pêgo-Fernandes

**Affiliations:** I Member of Staff, Pediatric Cardiac Surgery Unit, Instituto do Coração, Faculty of Medicine, Universidade de São Paulo, São Paulo, Brazil; Affiliate Professor, Department of Cardiopneumology, Faculty of Medicine, Universidade de São Paulo, São Paulo, Brazil.; II Chief Executive Officer, XenoBR, Member of the Transplant Coordination Department at Associação Brasileira de Transplante de Órgãos, São Paulo, Brazil; Researcher at the Donation and Transplantation Study Group, Universidade Federal de São Paulo, São Paulo, Brazil.; III Vice-director, Faculty of Medicine, Universidade de São Paulo, São Paulo, Brazil; Full Professor, Department of Cardiopneumology, Faculty of Medicine, Universidade de São Paulo, São Paulo, Brazil; Director of the Scientific Department, Associação Paulista de Medicina, São Paulo, Brazil.

 Xenotransplantation (XTx), the transplantation of organs, tissues, or cells from animals to humans, is emerging as a promising alternative to address the increasing global demand for transplantable organs. Over the past two decades, substantial progress has been made in transplantation practices worldwide, driven by advancements in healthcare infrastructure and the expansion of organ donation programs involving both living and deceased donors. Global data show a significant rise in transplant activity in regions such as the United States, Europe, China, and India, emphasizing a broader international effort to address organ shortages. Despite global advances in transplantation, considerable disparities in access persist, particularly in low- and middle-income countries, where limited infrastructure and socioeconomic inequalities continue to impede equitable care. Consequently, millions of patients continue to face barriers to life-saving transplants, highlighting the urgent need for international collaboration and strategic investments to expand access. 

 Brazil is recognized for maintaining one of the largest public transplant systems in the world. From preoperative preparation to lifelong posttransplant pharmacotherapy, transplantation constitutes one of the most resource-intensive interventions funded by the Unified Health System (*Sistema Único de Saúde*), which accounts for approximately 96% of all heart transplants performed in Brazil.^1^

 Despite significant advances in transplant techniques and public policies, the supply of organs still fails to meet the growing demand, resulting in increasingly long waiting lists, as evidenced by the Brazilian Transplant Registry.^2^ This scenario underscores the urgent need to guarantee fair access to transplantation, as established by the 1988 Federal Constitution and Law No. 8,080/1990,^3^ which defines health as a right of all and a duty of the State. In the context of a shortage of human organs and increasing demand for new therapeutic alternatives, XTx has emerged as a promising and potentially viable solution. 

 The history of XTx closely follows the overall development of transplantation. The earliest reports of such procedures involve tissue and cell transplants, rather than organ transplants.^4^ Initial experiments date back to the 17^th^ century, most notably the xenotransfusions conducted by Jean-Baptiste Denis in 1667 using the blood of a lamb.^5^ Another significant milestone occurred in 1838, when Richard Sharp Kissam performed the first corneal XTx by transplanting porcine corneal tissue into a human patient.^6^ Throughout the 19 ^th^ and 20^th^ centuries, advances in vascular anastomosis techniques—pioneered by Mathieu Jaboulay and Alexis Carrel—enabled a series of experimental attempts that involved organs from several species, including nonhuman primates (NHP; baboons and chimpanzees), pigs, rabbits, and sheep. However, these experiments were largely unsuccessful, primarily due to the occurrence of hyperacute rejection and other severe immunological complications.^4,7^

 The advent of modern immunosuppression in the 1960s, fueled by the discoveries of Peter Medawar and the introduction of drugs such as 6-mercaptopurine, marked a new era, enabling significant progress in both allotransplantation and XTx.^4^ From that point forward, the first clinical reports of cardiac XTx in humans began to emerge. Among the key milestones: 1964 – chimpanzee heart, by James Hardy, in Jackson (USA), with a survival time of 90 min;^8^ 1968 – porcine heart, by Donald Ross, in London (UK), with a survival time of 4 min;^9^ 1968 – sheep heart, by Denton Cooley, in Austin (USA), with a survival time of 10 min;^10^ 1969 – chimpanzee heart, by Marion, in Lyon (France), with immediate death;^11^ 1977 – baboon heart (patient 1) and chimpanzee heart (patient 2), by Christiaan Barnard, in Cape Town (South Africa), with survival times of 5.5 h and 4 days, respectively;^12^ 1984 – baboon heart, in the iconic "Baby Fae" case, by Leonard Bailey, in Loma Linda (USA), with a survival time of 20 days;^13^ 1992 – porcine heart, by Zbigniew Religa, in Sosnowiec (Poland), with a survival time of 23 h;^14^ and 1996 – porcine heart, by Dhani Ram Baruah, in Sonapur (India), with a survival time of 7 days.^15^ Notably, no lung XTx has ever been performed. 

 The main barrier—hyperacute rejection—remained insurmountable, resulting in survival times ranging from several minutes to a few days. During the 1990s, research progressed toward a more comprehensive understanding of the innate and adaptive immune systems, complement cascade, coagulation pathophysiology, and inflammatory responses.^7^

 Due to the unsuccessful use of NHP hearts for cardiac transplantation, pigs emerged as the most promising donor species for several reasons: their hearts closely resemble human hearts in size and anatomy; their genetic similarities to humans facilitate precise genetic modifications; they have favorable reproductive characteristics and husbandry practices; they pose a lower relative risk of infections; and, from an ethical and moral standpoint, their use is generally considered more acceptable than that of NHPs. However, due to significant evolutionary differences between pigs and primates, porcine organs transplanted into NHPs and humans undergo hyperacute rejection. A key factor in such rejection is the presence, in humans, of naturally occurring preformed antibodies against carbohydrate antigens expressed on the surface of porcine cells.^16,17^

 A major breakthrough in preventing hyperacute rejection was identifying the carbohydrate alpha-galactose-1,3-galactose linkage as the main xenoepitope responsible for triggering this immune response. After this discovery, genetically modified pigs lacking the gene for the enzyme alpha-1,3-galactosyltransferase were created, leading to the first successful experiments and opening a new chapter for XTx using genetically modified pigs.^16^

 The deletion of porcine genes related to immune regulation and thrombosis pathways led to further advancements in the field of XTx. In 2012, the introduction of the CRISPR/Cas9 gene-editing tool further facilitated the development of safer porcine donor lines by enabling the removal of porcine endogenous retroviruses.^18^

 On January 7, 2022, a genetically modified porcine heart, developed by United Therapeutics, was successfully transplanted into a 57-year-old patient with end-stage heart failure at the University of Maryland Medical Center, United States. The patient had been deemed ineligible for conventional advanced heart failure therapies. The patient died on March 8, 2022.^4^

 While early clinical initiatives beginning in the 1960s focused on heart transplantation, renal XTx has progressively gained broader acceptance, with accumulating clinical experience. This shift in preference is due to the technical challenges associated with cardiac transplantation using genetically modified porcine hearts, which contrasts with the following factors favoring renal XTx: (1) greater tolerance of the kidneys to temporary dysfunction (functional resilience);^19^ (2) anatomical and physiological compatibility of porcine kidneys with human systems;^20^ (3) lower immediate clinical impact of graft failure,^21^ including greater ease in removing the transplanted organ (graft nephrectomy); (4) a broader and more accumulated knowledge of renal immunology from preclinical models since the 1990s, particularly studies including porcine kidneys implanted into NHP, as well as the development of immunosuppressive therapies more precisely tailored to the renal environment;^18^ and achieved (5) faster progress in genetic modifications targeting kidney function. For example, deleting genes such as GGTA1, CMAH, and B4GALNT2, along with adding human genes like CD46, thrombomodulin, and CD47, has enhanced immunologic and functional performance, especially in the kidneys, leading to promising and potentially transformative clinical outcomes in humans.^19^ Therefore, choosing the kidney as the first solid organ for clinical XTx trials results from a combination of clinical, immunological, and operational factors that make it safer and more predictable for trials than vital organs lacking effective artificial support.^19,20^ This decision improves ethical and regulatory acceptability while enabling gradual and sustainable progress in the field of clinical XTx. In Brazil, promising initiatives are being led by experienced research groups, notably including renowned professionals such as Silvano Raia, Mayana Zatz, Rodrigo Vianna, and Jorge Kalil. The Brazilian project aims to establish a robust XTx platform, integrating sequential phases including advanced genetic engineering, embryo fertilization and cloning, management and development of genetically modified pigs, preclinical evaluations, and experiments involving brain-dead human subjects, culminating in clinical trials projected to begin in 2027. The goal is to obtain regulatory approval from the Brazilian Health Regulatory Agency (*Agência Nacional de Vigilância Sanitária*) by 2029. 

 Central aspects of the project include technical and scientific advances, as well as a strong focus on infrastructure dedicated to animal welfare. Strict protocols follow the standards established by the National Council for the Control of Animal Experimentation (*Conselho Nacional de Controle de Experimentação Animal*), with specialized animal care aimed at minimizing suffering and guaranteeing the highest health standards for the animals involved. 

 From an ethical and bioethical standpoint, XTx raises complex issues ranging from patient autonomy to clear criteria for candidate selection for the procedure. The importance of developing detailed and transparent informed consent forms, as well as guaranteeing justice and equity in access to XTx for all patients across both the public and private healthcare systems, remains a constant concern. 

 In the global context, the World Health Organization recommends the establishment of national regulatory bodies specifically dedicated to supervising XTx activities. These institutions must guarantee that all procedures are conducted in accordance with the highest ethical and safety standards. 

 The potential for clinical trials including solid organ XTx has been a topic of extensive debate. The cardiac xenotransplants performed in living human recipients at the University of Maryland sparked debate over the feasibility of initiating clinical trials for cardiac XTx. Several countries are currently deliberating whether—and how—to move forward with such trials. One example is the recent creation of the Medical Porcine Development Association in Japan, which reflects the growing interest of the local scientific community in XTx. The association aims to develop new medical care protocols and improve technologies for producing pigs intended for clinical use.^22^ In China, a major milestone was achieved with the first clinical liver XTx: a genetically modified auxiliary porcine liver was transplanted into a 71-year-old living patient as an approach to avoid small-for-size syndrome following extensive resection for hepatocellular carcinoma.^23^

 The field of XTx offers opportunities that extend beyond whole-organ replacement, with several interspecies applications already demonstrating promising clinical results. A notable example is implanting dopamine-producing cells derived from pigs into the substantia nigra of patients with Parkinson’s disease, with the goal of functional cell replacement. Additionally, the effectiveness of xenografts has been proven in various therapeutic settings, such as using porcine skin for temporary wound coverage, corneal transplantation to restore vision, and implanting porcine pancreatic islets as an experimental treatment for diabetes mellitus.^4^ Although further studies and regulatory discussions on clinical trials involving solid organ XTx are still needed, the procedures already being performed in living human recipients worldwide have increased global interest and scrutiny. XTx has become a realistic and promising treatment option, as long as it is conducted with scientific integrity, ethical responsibility, and strict regulatory oversight, always prioritizing human well-being and animal welfare. 

 As Elmi Muller emphasized in her opening address at the 30^th^ International Congress of The Transplantation Society (Istanbul, September 2024): "The future of transplantation depends not just on innovation but also on our commitment to ensuring that advancements reach all corners of the globe—ethically, sustainably, and equitably. Together, let us commit to a future where transplantation is not a privilege but a fundamental right—accessible, safe, and sustainable for all."^24^

 This perspective reinforces the need to prioritize XTx within the national research agenda in Brazil, including both basic and clinical sciences. 
